# Vital role for the *Plasmodium* actin capping protein (CP) beta-subunit in motility of malaria sporozoites

**DOI:** 10.1111/j.1365-2958.2009.06828.x

**Published:** 2009-08-24

**Authors:** Markus Ganter, Herwig Schüler, Kai Matuschewski

**Affiliations:** 1 Department of Parasitology, Heidelberg University School of Medicine69120 Heidelberg, Germany; 2 Parasitology Unit, Max Planck Institute for Infection Biology10117 Berlin, Germany; 3 Structural Genomics Consortium, MBB, Karolinska Institute17177 Stockholm, Sweden

## Abstract

Successful malaria transmission from the mosquito vector to the mammalian host depends crucially on active sporozoite motility. Sporozoite locomotion and host cell invasion are driven by the parasite's own actin/myosin motor. A unique feature of this motor machinery is the presence of very short subpellicular actin filaments. Therefore, F-actin stabilizing proteins likely play a central role in parasite locomotion. Here, we investigated the role of the *Plasmodium berghei* actin capping protein (*Pb*CP), an orthologue of the heterodimeric regulator of filament barbed end growth, by reverse genetics. Parasites containing a deletion of the CP beta-subunit developed normally during the pathogenic erythrocytic cycle. However, due to reduced ookinete motility, mutant parasites form fewer oocysts and sporozoites in the *Anopheles* vector. These sporozoites display a vital deficiency in forward gliding motility and fail to colonize the mosquito salivary glands, resulting in complete attenuation of life cycle progression. Together, our results show that the CP beta-subunit exerts an essential role in the insect vector before malaria transmission to the mammalian host. The vital role is restricted to fast locomotion, as displayed by *Plasmodium* sporozoites.

## Introduction

The actin-based microfilament system drives motile processes, such as cell motility, cytokinesis and vesicle transport in eukaryotic cells. These processes require dynamic interconversion of pools of monomeric and filamentous actin (G- and F-actin respectively), regulated by a large number of accessory proteins (Carlier and Pantaloni, 2007). Capping protein (CP) is a heterodimeric protein that controls assembly at the barbed (fast growing) end of the actin filament in non-muscle cells (Wear and Cooper, 2004; Cooper and Sept, 2008). Its skeletal muscle variant, CapZ, links barbed ends of filaments to the Z-line. CP is a central component of actin polymerization-driven cell motility, as it restricts growth of a subset of filaments thereby allowing fast, directed polymerization from a pool of unpolymerized actin. It is enriched in the periphery of motile cells, where filament growth pushes the cell envelope forward (Amatruda and Cooper, 1992). CP is necessary for *in vitro* reconstituted actin-based motility (Loisel *et al*., 1999). In *Saccharomyces cerevisiae*, *CP* null mutants are viable but display disorganized actin patches (Amatruda and Cooper, 1992). These loss-of-function mutants are synthetic lethal with null mutants of the filament cross-linking protein fimbrin (Adams *et al*., 1993). CP is also a component of the dynactin complex, where it binds to the barbed end of the actin-related protein 1 (Arp1) minifilament (Schafer *et al*., 1994). *CP* gene inactivation in *Dictyostelium*, *Drosophila* and mouse, resulted in increased length and bundling of actin filaments, excessive ruffling, and loss of lamellipodia and explosive formation of filopodia respectively (Hug *et al*., 1995; Rogers *et al*., 2003; Mejillano *et al*., 2004). These reverse genetic approaches demonstrated a direct role of *CP* in actin-based motility. Genetic work in *Drosophila* further established an essential function of the barbed end-capping protein in early embryogenesis of a multicellular organism (Hopmann *et al*., 1996).

In this study, we characterized the cellular role of *CPβ* in the unicellular eukaryote *Plasmodium*, the causative agent of malaria. Malaria remains the most important vector-borne infectious disease and particularly affects children in sub-Saharan Africa (Guinovart *et al*., 2006). During the complex life cycle in the mosquito vector and vertebrate host, the parasite follows a developmental program with alternating intracellular transformation and replication phases that lead to the formation of invasive stages. These stages, i.e. merozoites, ookinetes and sporozoites, are highly specialized for recognition of and entry into host cells, namely erythrocytes, the mosquito midgut epithelium and mammalian hepatocytes respectively. All malaria-associated symptoms and pathology originate exclusively from the pathogenic asexual red blood cell phase of the parasite life cycle. *Plasmodium* invasion differs fundamentally from receptor-mediated endocytosis, a hallmark of bacterial and viral host cell entry mechanisms (Sibley, 2004). Malaria and related parasites, such as *Toxoplasma gondii*, employ their own actin/myosin motor machinery to propel themselves into the host cell (Keeley and Soldati, 2004; Sibley, 2004). In addition, actin-based motility drives parasite locomotion and transmigration *en route* to the final target cell. Understanding the underlying molecular mechanisms has important implications for future malaria intervention strategies in order to target multiple stages and species simultaneously.

The actin motor machinery of the parasite features the short tailless motor MyoA (Meissner *et al*., 2002), tethered to the inner membrane complex by accessory proteins (Bergman *et al*., 2003), and very short polymers of actin that are linked to thrombospondin-related anonymous protein (TRAP)/MIC2-family invasins via aldolase (Buscaglia *et al*., 2003; Jewett and Sibley, 2003) and possibly other proteins. This arrangement mediates gliding on the substratum, apparently by moving F-actin–receptor complexes from the apical tip backwards along the parasite's longitudinal axis (Sibley, 2004; Schüler and Matuschewski, 2006a). The regulation of this motor machinery remains elusive. *Plasmodium* and related Apicomplexa encode only a fraction of the conventional microfilament regulators, with many protein families missing entirely (Baum *et al*., 2006; Schüler and Matuschewski, 2006b). Given the intrinsic instability of parasite actin polymers (Sahoo *et al*., 2005; Schmitz *et al*., 2005; Schüler *et al*., 2005) F-actin end-capping proteins are expected to be required for both orchestrated F-actin assembly and sustained filament stability. In *Plasmodium* genomes, two formin homology domains and two capping protein subunits can be identified, while WASP homology domains, an Arp2/3 complex and gelsolin-related proteins are apparently absent (Gordon and Sibley, 2005; Schüler and Matuschewski, 2006b).

*Plasmodium* and other apicomplexan parasites contain single copies of both CP subunits in their genomes. We previously identified *CPβ* in a screen for transcripts that are upregulated during maturation of sporozoite infectivity, indicating that *CPβ/UIS17* (upregulated in infectious sporozoites gene 17) plays a particularly important role during malaria transmission from the mosquito to the mammalian host (Matuschewski *et al*., 2002). Notably, infectious sporozoites are the only *Plasmodium* stages that display fast (1–3 μm s^−1^) gliding motility (Matuschewski and Schüler, 2008). In this study, we could identify an essential function for *CPβ* in sporozoite gliding locomotion and, as a consequence, colonization of *Anopheles* salivary glands. Our data suggest that F-actin capping by CP is vital for fast locomotion, a property of *Plasmodium* sporozoites that ensures efficient transmission to the vertebrate host.

## Results

### *Plasmodium* capping protein

Protein sequences of the putative beta capping protein subunits from different *Plasmodium* species (PFE0880c and PB000641.00.0 for *Plasmodium falciparum* and *P. berghei* CPβ respectively) share around 25% sequence identity with those of yeast, chicken and human (Fig. S1A). These genes are among the most divergent within the family (Cooper and Sept, 2008). However, the majority of the key residues stabilizing the heterodimer (Yamashita *et al*., 2003) and those implicated in actin binding (Barron-Casella *et al*., 1995; Yamashita *et al*., 2003; Narita *et al*., 2006) are present (Fig. S1). The *Plasmodium* genomes also encode the corresponding putative capping protein alpha-subunit (*CPα*).

**Fig. 1:**
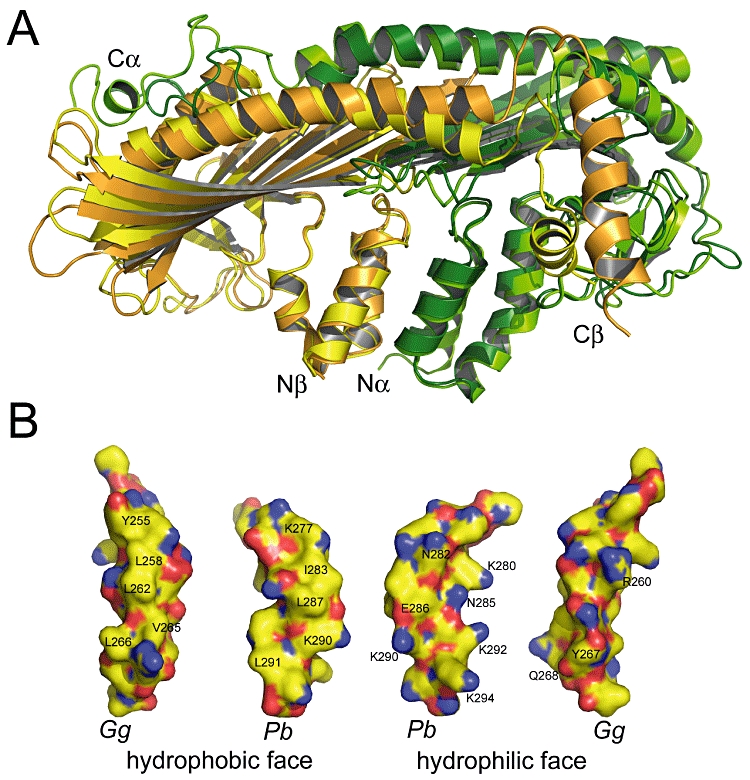
Homology model of *Plasmodium* capping protein (CP). A. Superposition of a structural model of the *Plasmodium berghei* CP heterodimer onto the crystal structure of the chicken CapZ heterodimer. The alpha-subunits of *P. berghei* and chicken CP are shown in light and dark green, and the beta-subunits of *P. berghei* and chicken CP are shown in yellow and gold respectively. The positions of the N-and C-termini are indicated. B. Amphipathic characters of the C-terminal tentacle extensions of parasite (*Pb*) and chicken (*Gg*) CPβ. Key side-chains contributing to the amphipathic character are indicated.

In order to evaluate the likelihood of these gene products being *bona fide* heterodimeric actin capping proteins we modelled their three-dimensional structure with SWISS-MODEL (Guex and Peitsch, 1997) using the chicken CapZ crystal structure as a template. A high fidelity model with < 1% Ramachandran outliers was readily generated (Fig. 1). In the *P. berghei* CPα/β homology model (Fig. 1), conserved side chains maintain the overall architecture as well as the interactions across the subunit interface, much as previously described for CapZ (Yamashita *et al*., 2003).

The C-terminal ‘tentacle’ extension of CPβ is implicated in actin binding by folding into an amphipathic α-helix that binds to a hydrophobic patch on actin (Narita *et al*., 2006). An additional function of the amphipathic α-helical C-termini of both CP subunits may be negative regulation of CP by binding of the tentacles through other proteins (Wear and Cooper, 2004). The modelled C-terminal tentacle of *Plasmodium* CPβ has a similar amphipathic character (Fig. 1B). Interestingly, it is about twice as long as that of chicken CapZβ, which is likely an adaptation to the structure of the parasite actin filament. In summary, our structural model is consistent with a heterodimeric *Plasmodium* capping protein that functions in actin polymer regulation.

**Fig. 2:**
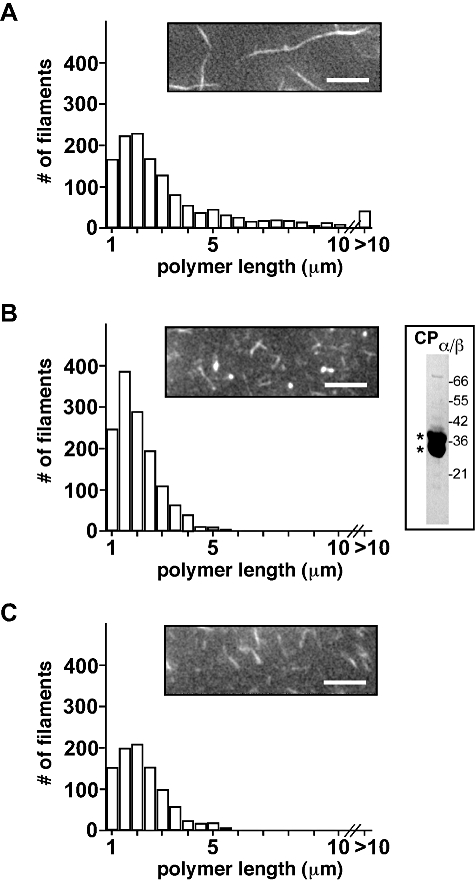
Recombinant *P. berghei* CP exhibits capping activity *in vitro*. Representative images of rhodamine-phalloidin-stained filaments and quantifications of length distribution of actin filaments alone (A) or in combination with 0.1 μm recombinant *P. berghei* CP (B) and 0.5 nm gelsolin (C) respectively. Polymers were imaged, their length determined (*n* = 1318; *n* = 1369; and *n* = 946 for control, *Pb*CP and gelsolin respectively), and polymers were sorted into 0.5 μm bins. The plotted length distributions of polymers between 0.5 and 20 μm is shown. Polymers shorter than 0.5 μm and longer than 20 μm were omitted from the analysis. The average microfilament lengths are 3.1 and 1.7 μm in (A) and (B) respectively. The insert in (B) shows the purified recombinant *Pb*CP protein used in this assay; asterisks indicate the positions of the CPα (upper) and CPβ (lower) signals. Bars represent 5 μm.

To confirm that *Plasmodium* CP has actin capping activity we employed fluorescence microscopy of actin filaments (Fig. 2). Coexpression of the two *Plasmodium* capping proteins in *Escherichia coli* and co-purification of the α/β subunits resulted in highly purified *P. berghei* CP (Fig. 2B). Since *Plasmodium* actin forms only very short filaments (Schmitz *et al*., 2005; Schüler *et al*., 2005), we employed heterologous non-muscle β-actin to test purified proteins for capping activity *in vitro*. Upon addition of *Pb*CP the length distribution of microfilaments shifted towards shorter filaments due to the barbed end capping activity of CP (Xu *et al*., 1999). *Pb*CP was able to reduce the average length of polymers by half (Fig. 2). We conclude that *Pb*CP displays *bona fide* F-actin capping activity *in vitro*.

**Fig. 3:**
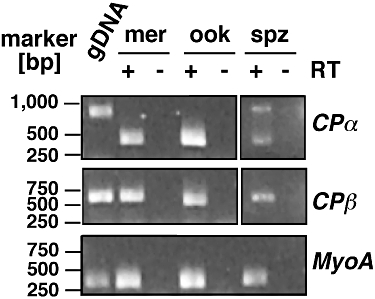
*Plasmodium CP* is expressed in all motile parasite stages. Presence of *CPβ*, *CPα* and the unconventional class XIV myosin *MyoA* was tested for transcript expression in three invasive *Plasmodium* stages: merozoites (mer) that invade erthrocytes, ookinetes (ook) that penetrate the mosquito midgut and sporozoites (spz) that invade the mammalian liver. *CPα* contains eight introns that distinguish amplification from gDNA and cDNA. No amplification is detectable in control reactions lacking reverse transcriptase (−RT).

In order to study the cellular role of *Plasmodium CP* for parasite propagation, we first tested transcript expression of both subunits in the three extracellular and invasive parasite stages, namely blood-stage merozoites that enter host erythrocytes, ookinetes that traverse the mosquito midgut epithelium and sporozoites that invade host hepatocytes (Fig. 3). As expected, the two genes are expressed in all motile stages similar to the motor protein *MyoA*. This expression pattern suggested important functions of *Plasmodium CP* in multiple extracellular life cycle stages.

### Generation of *cpβ(−)* parasites

We focused on the *in vivo* roles of *CPβ/UIS17* for parasite development in the two hosts. Reverse genetics in the rodent malaria model parasite *P. berghei* is particularly suited to study gene functions during *Plasmodium* life cycle progression. Initially, we targeted the endogenous *PbCPβ* locus with an integration vector that disrupts the corresponding open reading frame. Successful stable insertion of the integration plasmid suggested that *CPβ* is dispensable for propagation of the pathogenic blood stages (data not shown).

**Fig. 4:**
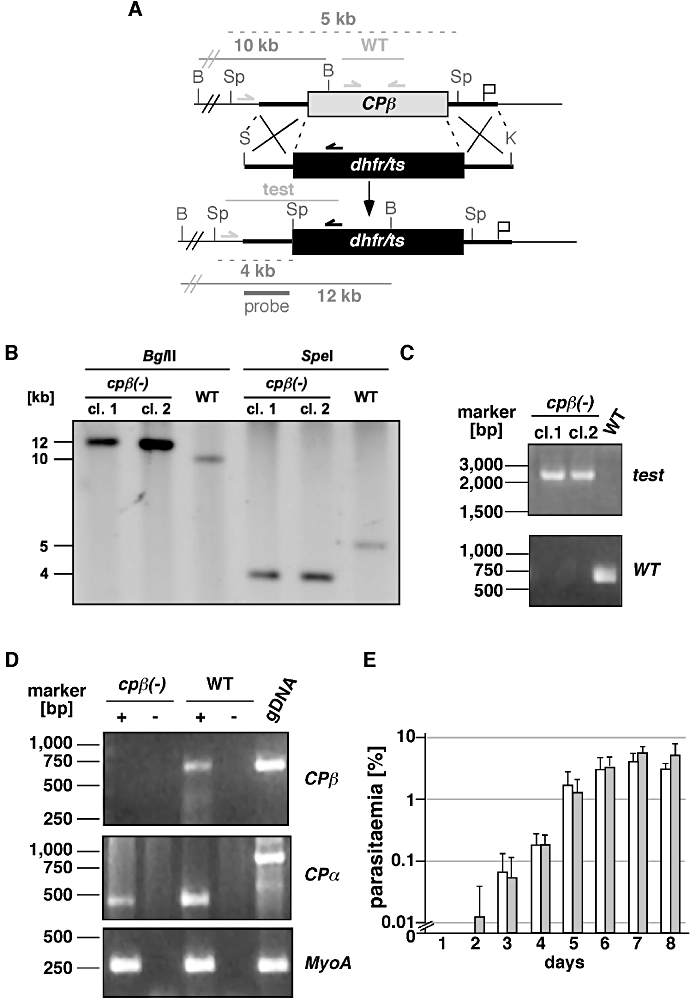
Targeted gene disruption of *P. berghei CPβ*. A. Replacement strategy to generate the *cpβ*(*−)* parasite. The wild-type (WT) *CPβ* genomic locus is targeted with a SacII/KpnI-linearized replacement plasmid containing the 5′ and 3′ untranslated regions of the *CPβ* open reading frame (ORF) and the *Toxoplasma gondii dhfr/ts*-positive selectable marker. Upon a double-cross-over event the *CPβ* ORF is replaced by the selection marker. Replacement-specific Southern fragments and test primer combinations are indicated by arrows, and expected fragments are shown as lines. B, BglII; Sp, SpeI; S, SacII; K, KpnI. B. Diagnostic Southern blot analysis of two clones and WT parasites. The fragments recognized by the 5′ probe in the BglII digest are 9.96 and 12.0 kb for the WT and *cpβ*(*−)* parasites respectively. Similarly, in the SpeI digest, the probe hybridizes to a 5.0 kb band in WT and a 3.98 kb band in *cpβ*(*−)* parasites. C. Replacement-specific PCR analysis. Confirmation of the predicted gene targeting is achieved by specific primer combinations that can only amplify a signal from the recombinant locus. Black and grey arrows in (A) indicate primers that hybridize to regions in the plasmid backbone and within and outside the *CPβ* ORF respectively. A WT-specific PCR reaction confirms the absence of residual wild-type parasites in the two clonal *cpβ*(*−)* populations. D. Depletion of *CPβ* transcripts in *cpβ*(*−)* parasites. cDNA from wild-type and *cpβ*(*−)* clone 1 merozoites was amplified at 35 PCR cycles. Note the absence of a *CPβ*-specific signal compared with a transcript control (*MyoA*). E. *cpβ*(*−)* parasites develop normally in the mammalian host. Asexual blood-stage development was determined by i.v. injection of 1000 infected erythrocytes. Parasitaemia of recipient animals (*n* = 5) was determined by daily quantification of Giemsa-stained blood smears. White bars, WT; grey bars, *cpβ(*−*)*. Shown are the mean values (± standard deviation).

To confirm this unexpected finding and to generate a genetically stable *CPβ* loss-of-function parasite line, we constructed a targeting vector that contained the 5′ and 3′*CPβ* flanking regions separated by the mutant *T. gondii dhfr/ts* gene cassette as a positive selection marker (Fig. 4A). Upon transfection the target gene locus is replaced with the selection marker via a double homologous recombination event. We obtained a parental population that contained mixed parasites with the disrupted *cpβ(−)* and the wild-type (WT)*CPβ* locus (not shown). Next, we cloned parasite lines *in vivo* by serial dilution and intravenous injection into 15 NMRI mice as recipient animals. We obtained six clones: three WT, one mixed and two *cpβ(−)* lines. Genotyping of the *cpβ(−)* clones by Southern blot analysis (Fig. 4B) and replacement-specific PCR analysis (Fig. 4C) showed absence of the WT-specific signal, and successful disruption of the *CPβ* locus. RT-PCR analysis with poly (A)^+^ RNA from WT and mutant mixed blood stages confirmed the absence of *CPβ* transcripts in the *cpβ(−)* parasite lines (Fig. 4D). Successful generation of *CPβ* loss-of-function parasite lines thus confirmed that this gene is not vital for propagation of the pathogenic asexual blood stages.

To test whether mutant parasites display a growth defect during blood-stage multiplication we performed an *in vivo* growth assay by intravenous injection of 1000 parasite blood stages into mice, followed by daily parasitaemia counts (Fig. 4E). Proliferation of *cpβ(−)* parasites was indistinguishable from WT parasites suggesting that *CPβ* is not essential in the pathogenic blood stages.

### *cpβ(−)* parasites are impaired in ookinete invasion of the mosquito midgut

We then tested the mutant parasites for transmission to the *Anopheles* vector. A fraction of asexual blood stages eventually enter sexual development giving rise to female and male gametes that fuse in the mosquito midgut to form a motile zygote, the ookinete (Kooij and Matuschewski, 2007). Quantification of sexual stages in Giemsa-stained blood smears of infected mice showed that *cpβ(−)* parasites are not affected in commitment to form mature gametes (Table S1). Similarly, we could not observe a loss-of-function phenotype in *in vitro* formation of ookinetes, the stage that penetrates the mosquito midgut epithelium (Table S1). Together, these findings suggest that transmission from the warm-blooded host to the invertebrate vector does not rely on functional *CPβ*.

**Fig. 5:**
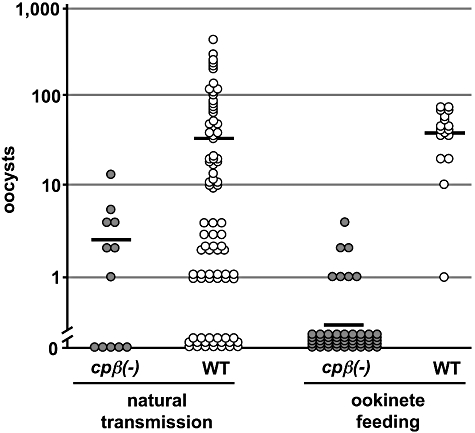
Loss of *CPβ* function precludes high oocyst densities. Quantification of oocyst numbers in *A. stephensi* mosquitoes that were infected either by natural transmission or by membrane feeding of *in vitro* cultured ookinetes. *cpβ*(*−)* and WT oocyst numbers and the average are shown as grey and white circles and bars respectively. *P*-values for *cpβ*(*−)* versus WT are in both cases *p* < 0.001.

**Fig. 6:**
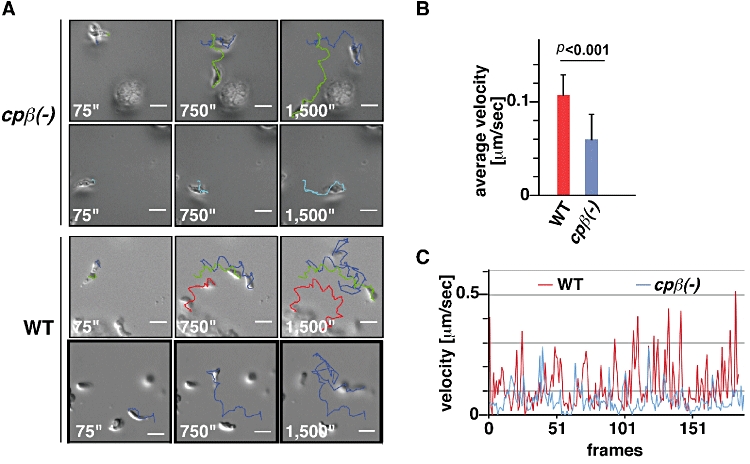
Impaired ookinete motility in *cpβ*(*−)* parasites. A. Time-lapse micrographs of cultured *cpβ*(*−)* (top) and WT (bottom) ookinetes. Shown are the 75, 750 and 1500 s time points. Both ookinete populations display productive motility as evidenced by the coloured traces. Scale bars, 10 μm. B. Quantification of ookinete motility. The average speed of WT (red) and *cpβ*(*−)* (blue) ookinetes was quantified from 12 and 25 representative ookinetes respectively. Shown are the mean values (± standard deviation). C. Representative quantification of velocities in WT and *cpβ*(*−)* ookinetes over a recording period of 50 min. One frame corresponds to 15 s.

When we counted oocyst numbers of WT and mutant parasites at day 10 after mosquito infection we could detect a significant phenotype that was, however, not complete (Fig. 5 and Fig. S2). The *cpβ(−)* parasites produce substantially fewer oocysts as compared with the isogenic WT. This finding was confirmed by membrane feeding of equal numbers of *in vitro* cultured ookinetes to *Anopheles* mosquitoes (Fig. 5), suggesting that the observed reduced ookinete infectivity does not depend on the presence of the peritrophic membrane that is formed after blood meal ingestion. Most importantly, mutant ookinetes that successfully penetrated the midgut epithelium are capable of producing mature midgut-associated oocysts (Fig. 5). This observation prompted us to test the ability of ookinetes to glide *in vitro* (Fig. 6 and Movies S1 and S2). In agreement with the impaired oocyst production rate we observed a reduction in ookinete motility. Quantitative analysis of ookinete velocities revealed an average speed of 3.7 (± 1.7) μm min^−1^ and 6.5 (± 2.0) μm min^−1^ for *cpβ(−)* and WT ookinetes respectively (Fig. 6B). Nevertheless, mutant ookinetes transiently displayed fast locomotion (Fig. 6C). Together, *cpβ(−)* ookinetes displayed productive motility and were apparently capable of reaching the midgut epithelium (Fig. 5). From these findings we conclude that *CPβ* plays an auxiliary role in ookinete penetration of the mosquito midgut epithelium.

### *cpβ(−)* sporozoites are non-motile and fail to invade salivary glands

To confirm the capacity of *cpβ(−)* parasites to form sporozoites we counted midgut-associated sporozoites at various time points after infection (Table 1). As expected, mutant parasites were able to produce substantial numbers of midgut sporozoites. In fact, the average number of midgut sporozoites was only moderately diminished as compared with the observed reduction in oocyst numbers (Fig. 5), consistent with a recent observation that *Plasmodium* development in the mosquito vector exhibits a strong density dependence, i.e. few oocysts produce disproportionally high numbers of sporozoites (Sinden *et al*., 2007).

**Table 1:** *cpβ(−)* parasites are deficient in salivary gland colonization.

Development in *Anopheles*	Day	*cpβ(−)* cl.2	*cpβ(−)* cl.1	WT
Mosquito infectivity	10	56% (± 27%) [3]	46% (± 23%) [7]	77% (± 23%) [3]
Oocyst sporozoites	14	4200 (± 1200) [3]	13 300 (± 10 000) [7]	62 500 (± 25 000) [3]
	17	3900 [1]	9000 (± 3300) [3]	N/D
	30	3300 [1]	6100 (± 2600) [3]	N/D
Haemocoel sporozoites	16	69 (± 98) [2]	160 (± 246) [3]	4700 (± 5300) [2]
Salivary gland sporozoites	17	0 [3]	44 (± 93) [6]	17 000 (± 8400) [7]
	30	0 [1]	33 (± 27) [3]	N/D

Shown are mean values (± standard deviation); number of independent experiments is given in square brackets. N/D, not determined.

When we followed sporozoite maturation we observed a dramatic loss-of-function phenotype (Table 1); *cpβ(−)* sporozoites lost their ability to invade salivary glands, the final target organ in the mosquito vector. Consistent with this finding, none of highly susceptible C57BL/6 mice or young Sprague/Dawley rats developed a malaria infection when *cpβ(−)*-infected *Anopheles* mosquitoes were used to infect naïve animals by natural mosquito bites (Table 2). This finding indicated an essential role of *CPβ* during sporozoite maturation, a prerequisite to complete the life cycle and infect the mammalian host (Matuschewski, 2006).

**Table 2:** *cpβ(−)* parasites are non-infectious to the mammalian host.

Parasite population	Inoculum^a^	Number of animals infected^b^	Pre-patency (days)^c^
WT	5 infect. mq.	8/8 (100%)	3.5
*cpβ(−)* cl.1	5 infect. mq.	0/2 (0%)	N/A
	10 infect. mq.	0/2 (0%)	N/A
	> 50 infect. mq.	0/2 (0%)	N/A
WT	100 000 i.v.	10/10 (100%)	5.4
*cpβ(−)* cl.1	50 000 i.v.	0/3 (0%)	N/A
	100 000 i.v.	0/5 (0%)	N/A
	880 000 i.v.	0/1	N/A

a Infection was either by intravenous (i.v.) injection of purified midgut sporozoites or by exposure to bites of infected mosquitoes (infect. mq.) at the numbers indicated.

b Highly susceptible animals, i.e. young Sprague/Dawley rats and C57BL/6 mice, were used for intravenous and by bite infections respectively. Animals were monitored by daily microscopic examination of Giemsa-stained blood smears for up to 28 days.

c The pre-patent period is the time until first detection of blood-stage parasites.

N/A, not applicable.

To test whether ablation of salivary gland invasion was the exclusive cause for the observed interruption of transmission, we isolated large numbers of midgut-associated sporozoites and injected them intravenously into susceptible mice (Table 2). Again, all infected mice stayed free of malaria. Next, we tested whether attenuation of *Plasmodium* life cycle progression in the *cpβ(−)* parasites can be reversed by inheritance of a WT copy during sporozoite formation. Previous work on the *P. berghei* LCCL/lectin adhesive-like proteins (LAPs) has established that heterozygous oocysts obtained by crossing mutant and WT parasites *in vivo* can rescue loss-of-function mutants (Raine *et al*., 2007). For the present study, we crossed *cpβ(−)* and WT parasites and genotyped the mixed parasites in comparison with clonal parasites before and after mosquito transmission. As predicted, in mixed inoculations the *cpβ(−)* genotype was recovered after life cycle completion (Fig. S3). These findings led us to conclude that the essential function of *Plasmodium CPβ* is restricted to the insect vector stages, and this deficiency can be rescued by transient complementation of one WT copy during oocyst development, where a heterozygous cell undergoes multiple rounds of replication prior to sporozoite budding.

**Fig. 7:**
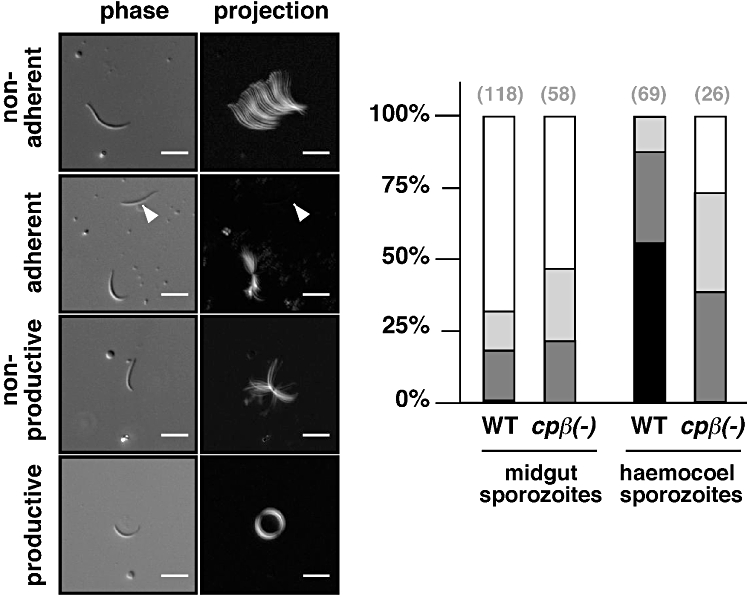
*CPβ* is essential for sporozoite gliding locomotion. Quantification of motility patterns in midgut- and haemocoel-associated sporozoites from WT and the *cpβ*(*−)* mutant (clone 1) (right). Shown is the percentage of non-motile, i.e. detached (white) and attached (light grey), sporozoites and sporozoites that display non-productive (dark grey) and productive (black) motility. In contrast to WT sporozoites that mature upon egress to the mosquito haemocoel *cpβ*(*−)* parasites remain non-gliding. Track projections (projections) and phase micrographs at time point 0 s (phase) of representative motility patterns of WT haemocoel sporozoites are shown on the left. On coated glass slides a proportion of WT sporozoites glide in circles at an average speed of 1–3 μm s^−1^ (represented by bottom panels). In marked contrast, *cpβ*(*−)* sporozoites display only non-productive motility patterns, such as bending, flexing and pendulum movement (represented by upper panels). Scale bars, 10 μm.

We finally studied sporozoite gliding locomotion. For this analysis, we isolated haemocoel sporozoites, the stage that is the most advanced in the mutant parasite lines. In marked contrast to WT sporozoites that perform continuous fast gliding (Fig. 7 and Movie S3), *cpβ(−)* sporozoites never displayed productive locomotion (Fig. 7 and Movie S4). Notably, bending and flexing, a microtubule-dependent form of non-productive motility (Vanderberg, 1975), was frequently observed, corroborating the viability of mutant sporozoites and supporting a distinct defect in forward locomotion. In agreement with defective sporozoite locomotion, we detected substantial numbers of midgut-associated sporozoites throughout the mosquito lifespan (Table 1), suggesting that active gliding motility is important for efficient sporozoite egress out of oocysts.

In conclusion, the *Plasmodium CPβ* subunit exerts a vital function during life cycle progression of the malaria parasite in the insect vector. Loss of *CPβ* function results in non-motile sporozoites that fail to colonize the mosquito salivary glands and, hence, cannot be transmitted to the vertebrate host.

## Discussion

The most important finding of our reverse genetics analysis of *Plasmodium CPβ* is a vital role for life cycle progression of the unicellular obligate intracellular malaria parasite. Our systematic phenotypic characterization of *P. berghei cpβ(−)* parasites revealed important cellular functions exclusively in locomotion of extracellular parasite stages. We establish that *CPβ* is essential for sporozoite gliding locomotion, a function that is gradually acquired during sporozoite maturation and required for subsequent transmission to the mammalian host (Matuschewski, 2006). This stage-specific vital role of *CPβ* correlates with fast motility, a feature observed only in mature, salivary gland-associated sporozoites. In analogy, we predict that the *T. gondii CPβ* protein performs essential functions for motility of tachyzoites, the fast-replicating stage that infects all nucleated cells.

Unexpectedly, *CPβ* is dispensable for asexual erythrocytic parasite growth, the life cycle phase that causes all malaria-related morbidity and mortality. Proliferation of *cpβ(−)* parasites was indistinguishable from WT parasites. Two actin-dependent processes appear to be central to the intra-erythrocytic cycle, i.e. endocytic haemoglobin uptake (Smythe *et al*., 2008) and erythrocyte invasion (Miller *et al*., 1979). Since a defect in haemoglobin uptake, the major amino acid source for the parasite, likely translates into delayed and/or decreased merozoite formation, we postulate that an actin capping activity, if any, is either redundant or mediated by proteins other than CPβ. Inhibitor studies established a requirement for parasite actin in merozoite invasion (Miller *et al*., 1979). In support of a non-vital role of *CPβ* in actin capping during this process *PbCPβ* transcripts are readily detectable in free merozoites. In a previous biochemical study three major F-actin binding proteins, i.e. HSP70 and two unknown proteins of 32 and 34 kDa, were isolated from *Plasmodium knowlesi* merozoites (Tardieux *et al*., 1998). The F-actin binding properties and the respective molecular sizes of the yet unidentified proteins are consistent with the heterodimeric CP protein. In addition, the formin-like protein 1 has been localized in the vicinity of the parasite restriction that forms upon cell entry, termed moving junction (Baum *et al*., 2008). Formin-dependent incorporation of G-actin monomers into the growing filament resembles a transient barbed end capping function that may partially compensate for the lack of *CPβ*. However, it is important to note that merozoite invasion into erythrocytes is typically completed within 30 s. Therefore, a reduced speed does not necessarily translate into a detectable alteration of parasite propagation, since the intra-erythrocytic life cycle phase of *P. berghei* takes 24 h. Hence, either *cpβ(−)* parasites may have a partial invasion defect that remains undetected *in vivo* or *CPβ* is dispensable for the motility of merozoites, which appear to lack the capacity to glide on substrates entirely.

Direct proof for a role of *CPβ* in cell motility comes from the intermediate phenotype observed in *cpβ(−)* ookinetes that display an overall reduced motility, while all other functions, including zygote formation and viability as well as subsequent sporogony, remain unaffected. Membrane feedings of *in vitro* cultured ookinetes excluded a defect in penetration of the peritrophic membrane, which is formed exclusively after a blood meal and requires secretory parasite proteins, such as chitinase, to be permissive for ookinetes.

The observed intrinsic instability of *Plasmodium* actin (Schmitz *et al*., 2005; Schüler *et al*., 2005) is expected to result in rapid microfilament depolymerization. Therefore, the cellular role of CP as an F-actin stabilizer may be particularly important in this model eukaryote. Barbed end capping by CP is essential for fast actin/myosin-dependent gliding locomotion, as displayed by mature infectious *Plasmodium* sporozoites. Incidentally, this forward motility displaying an average speed of 1–3 μm s^−1^ is among the fastest gliding locomotions on substrates for any eukaryotic cell (Vanderberg, 1975). We propose that barbed end capping by capping protein is vital for rapid microfilament turnover to achieve high speed actin-dependent locomotion *in vivo*.

## Experimental procedures

### Experimental animals

Animals were from Charles River Laboratories. All animal work was conducted in accordance with European regulations and approved by the state authorities (Regierungspräsidium Karlsruhe).

### Parasite transfection and genotypic analysis

For replacement of *PbCPβ* we employed primers *PbCPβ_*forI (5′-ATCCCCGCGGAGTACATGCAATATATACATATATATTCAATGC-3′; SacII site is underlined) and *PbCPβ_*revII (5′-ATAAGAATGCGGCCGCAATTTAGTTTTTTTATATGGTTATTTATTTATTACAG-3′; NotI site is underlined) for amplification of the 5′ flanking region, and *PbCPβ_*revIII (5′-CCCCAAGCTTGAATGCGATTTTAGGGGCCAATACAATTAGC-3′; HindIII site is underlined) and *PbCPβ_*revIV (5′-CGGGGTACCCGATTTTTTTTATTAATTCATCAAATTTTCCC-3′; KpnI site is underlined) for the 3′ flanking region, respectively, using *P. berghei* genomic DNA as template. Cloning into the *P. berghei* transfection vector (Thathy and Ménard, 2002) resulted in the plasmid p*PbCPβREP*. The targeting plasmid was linearized with SacII and KpnI, and parasite transfection, positive selection and parasite cloning were performed as described previously (Janse *et al*., 2006). Standard Southern blot analysis was performed with a commercial kit (DIG High Prime Labelling and Detection Starter kit II, Roche). For probe amplification we utilized primers *PbCPβ_*forI and *PbCPβ_*revII. We obtained two independent *cpβ(−)* clonal parasite populations that were phenotypically identical. Detailed analysis was performed with one representative clone.

### Transcript detection

For RT-PCR analysis, we isolated poly (A^+^) RNA using oligo dT-columns (Invitrogen). For cDNA synthesis and amplification, we performed a two-step PCR using oligo dT primers (Ambion) and subsequent standard PCR reactions, using gene-specific primers.

### Analysis of parasite development

*Anopheles stephensi* mosquito rearing and maintenance was carried out under a 14 h light/10 h dark cycle, 75% humidity and at 28°C or 20°C respectively. Blood-stage development was analysed *in vivo* in asynchronous infections using Naval Medical Research Institute (NMRI) mice. Gametocyte differentiation and exflagellation of microgametes were detected in mice before ookinete culture or mosquito feedings respectively.

Ookinete culture was conducted in RPMI 1640 medium with l-glutamine and 25 mM HEPES (Gibco) supplemented with 100 mM sodium bicarbonate; 100 μm hypoxanthine; 10% FCS; 50 μm xanthurenic acid; and 125 U ml^−1^ penicillin/streptomycin. For ookinete culture, we treated Theiler's Original (TO) mice with phenylhydrazine (1.2 mg per mouse) 24 h before infection. Infected blood was collected by cardiac puncture 4 days after infection and added to 10 volumes of culture medium. After incubation for 24 h at 20°C, erythrocytes were lysed for 20 min in ice-cold ammonium chloride solution (170 mM), and ookinetes were washed and re-suspended in HBSS. For artificial membrane feeding of ookinetes, cultured ookinetes were mixed with 1 ml of blood from a naïve mouse and applied to a temperature-regulated glass feeder.

Ookinete motility was analysed by time-lapse microscopy. Cultured ookinetes were purified, mixed with mos20 cells and imaged on a Zeiss Axiovert M200 microscope (Carl Zeiss, Göttingen, Germany). Images were captured using a CoolSnap HQ camera (Photometrics, Tucson, AZ, USA) and MetaMorph imaging software (Molecular Devices, Downingtown, PA, USA). Processing of all images was conducted utilizing the program ImageJ.

Sporozoites were dissected and analysed as described previously (Vanderberg, 1975). For determination of sporozoite infectivity, infected mosquitoes were dissected at days 14–30 after feeding. Sporozoites were liberated and injected intravenously at the numbers indicated into young Sprague/Dawley rats or C57BL/6 mice respectively. Patency was determined by daily examination of Giemsa-stained blood smears.

### Homology modelling

The homology model of the *P. berghei* CPα/β heterodimer was constructed based on sequence alignments with the respective chicken CapZ subunit sequences using SWISS-MODEL (Guex and Peitsch, 1997). The α-subunits are 23.8% identical and 42.3% similar, and the beta-subunits are 30.7% identical and 37.2% similar on the amino acid level. The model was superimposed onto the crystal structure of the chicken CapZ heterodimer (PDB entry 1izn). The beta-subunit tailpieces shown in Fig. 1B represent the sequences 250-PDNQKYKQLQRELSQVLTQRQI-271 (*Gg*CPβ) from crystal structure 1izn and SKGNIQNELKSKLKKK (*Pb*CPβ) from the homology model based on that structure.

### Recombinant protein expression and purification

Recombinant active *P. berghei* capping protein heterodimer was produced by coexpression of both alpha- and beta-subunits from the same expression plasmid as described previously for chicken CP (Soeno *et al*., 1998), employing the pET-Duet-1 vector system (Novagen). Expression of the recombinant capping protein subunits, a N-terminally hexahistidine-tagged *Pb*CPα and C-terminally S-tagged *Pb*CPβ, were induced using 0.5 mM IPTG. Recombinant capping proteins were purified from lysed bacterial pellets using HisTrap HP columns (GE Healthcare, Piscataway, NJ, USA) equilibrated with 20 mM Tris pH 8.0, 20 mM imidazole and 300 mM NaCl, 10% glycerol. After washing, proteins were eluted in 1 ml fractions with 20 mM Tris pH 8, 400 mM imidazole, 300 mM NaCl, 10% glycerol. Protein identity was confirmed by TOF-MS analysis. Capping protein concentrations were determined using the BCA Protein Assay Kit (Pierce) and stored as aliquots at −80°C. Non-muscle β-actin was purchased from Cytoskeleton (Frankfurt, Germany) and gelsolin from Sigma-Aldrich (Taufkirchen, Germany).

### F-actin assays

As described previously (Xu *et al*., 1999), samples of actin (5 μm) were induced to polymerize by addition of 1 mM MgCl_2_ and 0.15 M KCl, and incubated at room temperature for 2–3 h. Actin polymers were supplemented with 100 nm rhodamine-phalloidin (Invitrogen) and incubated for 15 min at room temperature on coverslips in the presence of purified recombinant *P. berghei* capping protein. Samples were mounted in Vectashield (Vector Laboratories, Burlingame, CA, USA) and imaged using a 100× Fluoroplan oil immersion lens on a Zeiss Axiovert M200 microscope (Carl Zeiss, Göttingen, Germany). Images were captured using a CoolSnap HQ camera (Photometrics, Tucson, AZ, USA) and MetaMorph imaging software (Molecular Devices, Downingtown, PA, USA).

Actin polymer length measurements were carried out using ImageJ software. Polymers were sorted into 0.5 μm bins and their length distributions plotted. Polymers shorter than 0.5 μm and longer than 20 μm were omitted from the analysis.
