# Supplementary material for: Vital role for the Plasmodium actin capping protein (CP) beta-subunit in motility of malaria sporozoites
Source: Mol Microbiol. 2009 Aug 24;74(6):1356–67. doi: 10.1111/j.1365-2958.2009.06828.x (PMC2810434; doi:10.1111/j.1365-2958.2009.06828.x)
Supplement: Supplementary file 5 [file mmi0074-1356-SD5.pdf]

Supplemental material

- **Suppl. Table I**
- **Suppl. Figures 1 to 3**
- **Suppl. Movies 1 to 4**

Supplemental Table I: ***cpβ(-)*** **parasites produce gametocytes and ookinetes**

		% Gametocytes[b]		
Experiment	Parasite[a]	Female	Male	*in vitro* ookinetes ($x10^6$)[c]
1	*cpβ(-)*	2.3 (± 1.1)	0.5 (± 0.4)	0.4 (± 0.2)
	WT	2.5	1.6	3.6
2	*cpβ(-)*	2.8 (± 0.3)	2.0 (± 1.2)	0.5 (± 0.05)
	WT	1.5	0.7	0.6
3	*cpβ(-)*	4.8 (± 1.8)	1.6 (± 0.8)	18 (± 12)
	WT	5.6	0.8	10

[a] Data are from triplicates and at least duplicates for the *cpβ(-)* and WT parasites, respectively.

[b] Gametocytes were counted by examination of Giemsa-stained blood smears on day of optimal exflagellation and are shown as percentage of total blood-stage parasites.

[C] Ookinetes were cultured overnight at ambient temperature and counted with a hemocytometer.

Legends to Suppl. Figures

Suppl. Figure 1. Sequence alignments of *Plasmodium* capping protein subunits. (A) Primary structure of *Plasmodium*, yeast, human and chicken CPβ. Amino acid sequence identities of the CP beta subunits are indicated as percentages of identical amino acid residues compared with the *P. berghei* sequences. (B) Alignment of CPβ sequences from *Plasmodium berghei* (*Pb*CPβ; XM_674749), *P.falciparum* (*Pf*CPβ; XM_001351679), *Saccharomyces cerevisiae* (*Sc*CPβ; X62630), human (*Hs*CPβ; AAI09243), and chicken (*Gg*CPβ; NM_205437). Strictly conserved amino acid residues are boxed in dark red.

Suppl. Figure 2. *cpβ(-)* parasites produce fewer oocysts. Representative phase contrast images of isolated *Anopheles stephensi* midguts that were either fed on a *cpβ(-)*- or a WT-infected mouse 10 days earlier. Mature oocysts are indicated by white arrowheads. Scale bars, 100 μm.

Suppl. Figure 3. Genotyping of *cpβ(-)/*WT crosses. (A) Rescue of the *cpβ(-)* mutant by a genetic cross with WT parasites. Mosquito feedings on mice infected with a mix of *cpβ(-)* and WT parasites (input) results in heterokaryotic polyploid oocysts, and, ultimately, in infective haploid *cpβ(-)* sporozoites. Midgut- and salivary gland-associated sporozoites were isolated and injected into susceptible young Sprague/Dawley rats. All animals became patent, and the parasites were genotyped by PCR. On the right the genotyping of the corresponding control clones (*cpβ(-)* and WT, respectively) are shown. Note that repeatedly *cpβ(-)* parasites were recovered, suggesting that WT-rescue during oocyst development is sufficient to overcome the sporozoite-specific transmission block. (B) Genotyping of sporozoites isolated

from naturally fed mosquitoes. Midgut (mg) and salivary gland (sg) sporozoites were isolated from *A. stephensi* mosquitoes fed on mixed *cpβ(-)/*WT infections at days 14, 30, and 17, respectively. After isolation of genomic DNA, *cpβ(-)*- and WT-specific signals were amplified with specific primer combinations. DNAs from clonal parasites were added as controls. These data show successful colonization of the haploid *cpβ(-)* sporozoites in the presence of CPβ protein during sporogony. (C) Genotyping of blood stages from animals infected with sporozoites from ookinete-injected mosquitoes. Animals were infected either by mosquito bite or intravenous injection of salivary gland sporozoites from *A. stephensi* mosquitoes that were inoculated with a mixture of cultured WT and *cpβ(-)* ookinetes. DNAs from clonal parasites were added as controls. This control confirms the defect of *cpβ(-)* sporozoites that no longer complete the life cycle when fed as homozygous tetraploid ookinetes.

Ganter *et al.*, Suppl. Figure 1

A

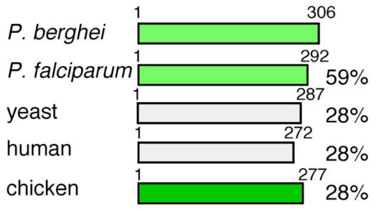

B

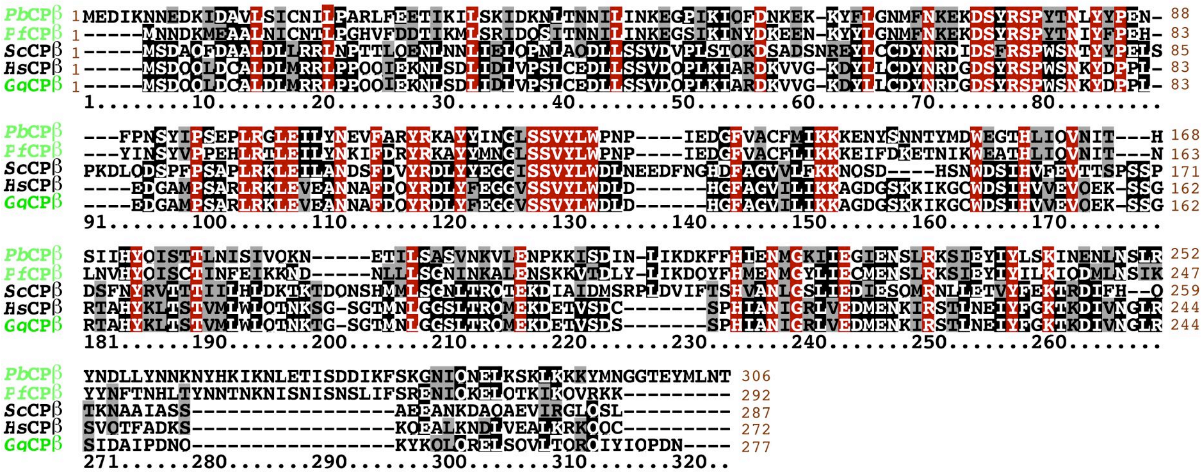

Ganter *et al*., Suppl. Figure 2

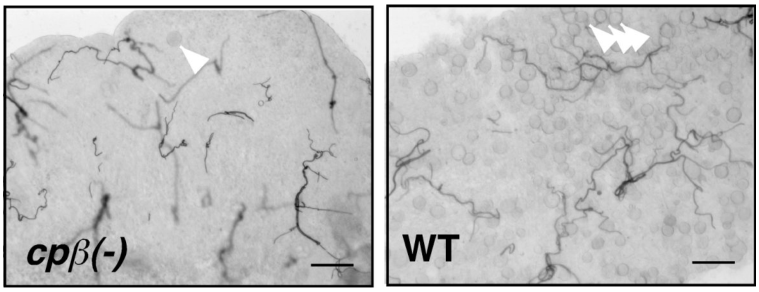

Ganter *et al.*, Suppl. Figure 3

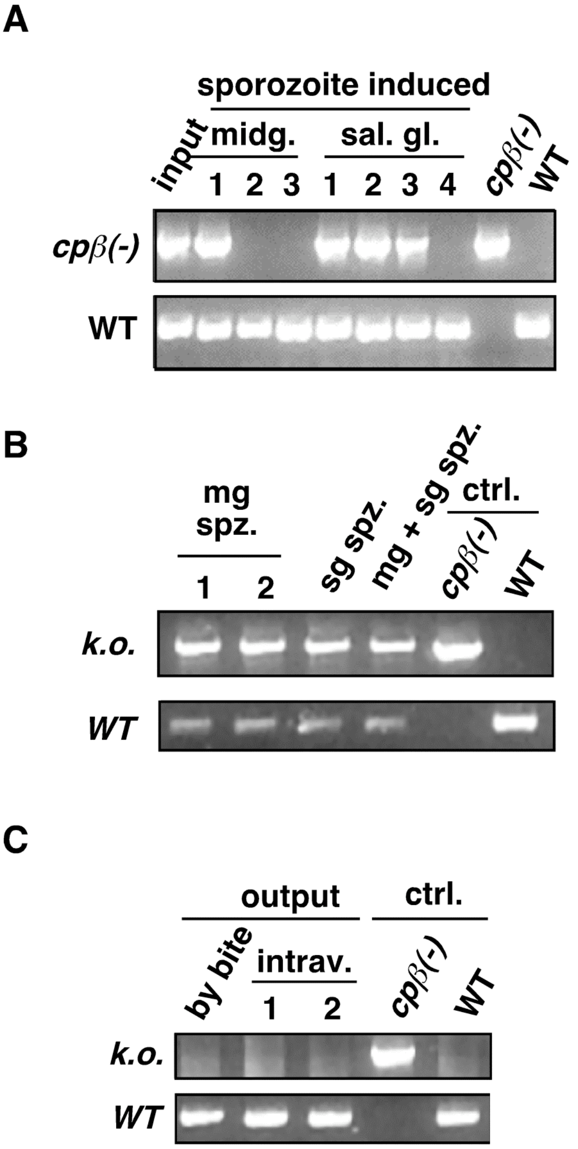

Legends to Suppl. Movies

Suppl. Movie 1. Gliding motility of *cpβ(-)* ookinetes on glass slides, co-incubated with MOS20 cells. Pictures were taken in 15 sec. intervals. Total time: 25 min. Scale bar, 10 μm.

Suppl. Movie 2. Gliding motility of *WT* ookinetes on glass slides, co-incubated with MOS20 cells. Pictures were taken in 15 sec. intervals. Total time: 25 min. Scale bar, 10 μm.

Suppl. Movie 3. Typical, continuous, and circular gliding locomotion of WT hemocoel sporozoites on glass slides, co-incubated with 3% bovine serum albumine. Pictures were taken in 2 sec. intervals. Total time: 100 sec. Scale bar, 10 μm.

Suppl. Movie 4. Non-productive gliding motility of *cpβ(-)* hemocoel sporozoites on glass slides, co-incubated with 3% bovine serum albumine. Pictures were taken in 2 sec. intervals. Total time: 100 sec. Scale bar, 10 μm.
